# Identification of a Nonsense Mutation in CWC15 Associated with Decreased Reproductive Efficiency in Jersey Cattle

**DOI:** 10.1371/journal.pone.0054872

**Published:** 2013-01-22

**Authors:** Tad S. Sonstegard, John B. Cole, Paul M. VanRaden, Curtis P. Van Tassell, Daniel J. Null, Steven G. Schroeder, Derek Bickhart, Matthew C. McClure

**Affiliations:** 1 Bovine Functional Genomics, United States Department of Agriculture, Agricultural Research Service, Beltsville, Maryland, United States of America; 2 Animal Improvement Programs Laboratories, United States Department of Agriculture, Agricultural Research Service, Beltsville, Maryland, United States of America; Wageningen UR Livestock Research, The Netherlands

## Abstract

With the recent advent of genomic tools for cattle, several recessive conditions affecting fertility have been identified and selected against, such as deficiency of uridine monophosphate synthase, complex vertebral malformation, and brachyspina. The current report refines the location of a recessive haplotype affecting fertility in Jersey cattle using crossover haplotypes, discovers the causative mutation using whole genome sequencing, and examines the gene’s role in embryo loss. In an attempt to identify unknown recessive lethal alleles in the current dairy population, a search using deep Mendelian sampling of 5,288 Jersey cattle was conducted for high-frequency haplotypes that have a deficit of homozygotes at the population level. This search led to the discovery of a putative recessive lethal in Jersey cattle on *Bos taurus* autosome 15. The haplotype, denoted JH1, was associated with reduced fertility, and further investigation identified one highly-influential Jersey bull as the putative source ancestor. By combining SNP analysis of whole-genome sequences aligned to the JH1 interval and subsequent SNP validation a nonsense mutation in *CWC15* was identified as the likely causative mutation underlying the fertility phenotype. No homozygous recessive individuals were found in 749 genotyped animals, whereas all known carriers and carrier haplotypes possessed one copy of the mutant allele. This newly identified lethal has been responsible for a substantial number of spontaneous abortions in Jersey dairy cattle throughout the past half-century. With the mutation identified, selection against the deleterious allele in breeding schemes will aid in reducing the incidence of this defect in the population. These results also show that carrier status can be imputed with high accuracy. Whole-genome resequencing proved to be a powerful strategy to rapidly identify a previously mapped deleterious mutation in a known carrier of a recessive lethal allele.

## Introduction

Approximately 400 Mendelian disorders have been identified in cattle species [1], and new conditions are periodically identified [e.g., 2–4]. Matings among carriers can be avoided when reliable tests for a disorder are available, but the importation and sale of semen and embryos from known carriers often is prohibited, reducing rates of genetic gain and diminishing profitability of farms and genetics companies merchandising semen and embryos. Economic impacts on producers may be small, such as when a recessive has a small effect on fertility, or large, as in the case of higher stillbirth rates or the death of adolescent cows.

Several studies have demonstrated the utility of dense SNP genotypes for identifying chromosomal regions associated with autosomal recessive conditions in livestock [e.g., 5–9]. Mutations underlying recessive disorders in cattle have successfully been identified using DNA sequencing [10], and the cost of DNA sequencing has decreased dramatically, dropping from an average of $500.00 per Mb in October 2007 to $0.09 per Mb in October 2011 [11]. A two-stage strategy based on SNP genotyping and next generation resequencing is, therefore, an attractive option for identifying causal variants. In the first step, SNP genotypes are used to identify animals that are carriers of the haplotype of interest. In the resequencing step, carrier animals are selected for full genome sequencing based on such factors as their relationship to the breed and the frequency of their haplotypes in the population. Sequences from these animal are then aligned to the target interval of the genome to identify potential sequence variants underlying the phenotype.

VanRaden et al. [12] recently reported the discovery of five haplotypes with deleterious effects on fertility in three breeds of dairy cattle, including one recessive in Brown Swiss cattle, three in Holsteins, and one in Jerseys. The Jersey haplotype (JH1) was localized to the region between 11 and 16 Mbp on *Bos taurus* (BTA) autosome 15, and was determined to have a significant, negative effect on fertility. Despite the relatively high carrier frequency (23.4%) in the population, no homozygous animals have been identified in the population, leading to the conclusion that JH1 is associated with early embryonic loss. The 5-Mb region identified using SNP genotypes was therefore, an ideal candidate for resequencing to identify the causal variant associated with this phenotype.

The objective of this study was to identify the DNA sequence variation associated with lethal haplotype JH1 using targeted resequencing of heterozygous animals and to identify the putative biological mechanisms underlying the JH1 phenotype.

## Materials and Methods

### JH1 Haplotype

The initial search for lethal recessives [12] identified JH1 as a 73-marker haplotype on BTA15 spanning about 5 Mb located from 11,439,502 to 16,147,383 on the UMD 3.1 map [13]. From the JH1 frequency and the mating pattern, 90 homozygotes were expected but none were observed. For each expected homozygote, four carrier-to-carrier matings are needed. Therefore, a total of 90*4 or 360 carrier matings should have been observed. The probability of observing no homozygotes by chance alone is equivalent to 0.75^360^ or 10^−45^.

The inheritance of JH1 was traced to just one source ancestor, Observer Chocolate Soldier (US registration number 596832) born in 1962 [14]. Soldier’s daughters produced 764 liters per year more milk than daughters of average bulls born in 1962 and, as a result, he sired 1,454 daughters. In addition, Soldier’s impact was further magnified by 107 sons and 715 grandsons used in artificial insemination to generate >50,000 granddaughters, and >200,000 great granddaughters. As a result, the frequency of JH1 heterozygotes rose rapidly initially and has stabilized at 20 to 25% since 1980. This illustrates how an animal that is a genetic outlier with respect to an economically important trait can influence the frequency of favorable and unfavorable alleles in a population.

### Fertility Records

Examination of 52,449 fertility records of JH1 heterozygous bulls mated to daughters of JH1 heterozygous bulls revealed a conception success rate of 33.3% vs. 37.0% for 290,373 contemporary matings of normal bulls to daughters of normal bulls [15]. The expected decrease in conception rate for a simple recessive was calculated from the mean conception rate for cows assuming a one-eighth mortality ratio [12], and was 4.6 percentage points (37/8) for Jerseys. However, the estimated interaction is less than the simple expectations because other terms in the model (main effects of sire and MGS and inbreeding regression) remove a portion of the expected effect. The presence of pedigree errors also probably accounts for differences between the expected and observed reductions in conception rate (Text S1). The reduction in conception rate of 3.7%±0.2% confirmed JH1 as a lethal recessive, in approximate agreement with the 4.6% reduction expected. Conception success rate was 36.3% in 57,523 matings of heterozygous bulls to daughters of normal bulls, with the slight reduction of 0.7%±0.2% potentially caused by heterozygous dams with maternal JH1 inheritance. Matings were coded as successes only if the pregnancy went to term.

Embryonic losses from JH1 appear to occur very early in gestation (<60 d). Analysis of 60-, 100-, and 140-day non-return rates, a measure of fertility defined as the proportion of cows that do not return to estrus at a fixed time after insemination, shows that pregnancy losses did not increase after 60 days post-insemination [14]. This suggests that most embryonic losses occur before 60 days in gestation. Examination of 1,612 stillbirth records from JH1 heterozygous bulls mated to daughters of JH1 heterozygous bulls revealed perinatal mortality (stillbirths and deaths in the first week of life) of 7.6%±0.8% vs. 8.0% for normal contemporaries, indicating no significant effect. This result is consistent with the early embryo loss.

### Haplotype Detection and Crossover Analysis

Crossover haplotypes contain part of the source haplotype and part of a non-source haplotype, and a descendant’s phenotype status may be unknown when crossovers occur. Crossovers were detected from genotypes by directly comparing progeny to parent haplotypes within the pedigree using findhap.f90 [16] from 7,200 Jerseys with 50 K genotypes. For each crossover, the last marker known to be from the first parental haplotype and the first marker known to be from the second parental haplotype are output. A gap may remain between those two markers if the parental haplotypes are identical in that region, some markers are not called, or both parents were heterozygous and could not be phased leading to an unknown crossover point. As few dams are genotyped, crossovers occurring in maternal ancestors are often undetected.

Fine mapping was accomplished by checking for animals with both the original JH1 haplotype and a crossover haplotype [15]. Regions that were homozygous for a section of the source haplotype were removed from consideration of harboring the causative JH1 mutation. For example, if a live animal received the original JH1 haplotype from one parent and the left 20 markers of the JH1 haplotype from the other parent, the region containing those 20 markers were removed from consideration.

### Sequencing Analysis and SNP Discovery

Whole genome re-sequencing of animals was done on the Illumina HiSeq 2000 according to the manufacturer’s protocols (Illumina Inc., San Diego, CA). Paired-end libraries were prepared from 5 µg of genomic DNA purified from semen and sheared to about 300 bp using a Covaris S1 ultra-sonicator (Covaris, Woburn, MA). Bulls were selected for sequencing to maximize diversity of the alternative (non-JH1 carrier) haplotype and minimize relationships among animals with the JH1 haplotype, and semen was provided by the Cooperative Dairy DNA Repository (Beltsville, MD). Each library was loaded to a single channel on a flow cell (no pooling), and sequence data was generated as 2×100 bp reads. Initial sequence processing and base-calling was performed using Illumina CASAVA v.1.8.2 pipeline.

Sequence reads for each animal were aligned to the UMD 3.1 *Bos taurus* genome assembly using BWA [17]. All reads were treated as 100 bp paired-end reads and alignments allowed no more than 5 mismatches. Greater than 97% of all reads from the two high coverage groups (8 samples) were successfully mapped with more than 95% having proper mate pair orientation. The lower coverage samples mapped less well with 90% of reads mapping and 89% having proper mate pairing.

The aligned reads were used to identify sequence variations within the suspect region of BTA15. The SAMtools utility mpileup [18], [19] was used to identify and extract all SNPs and INDELs from BTA15 in the region of 11,430,000 to 16,150,000. Variants in the region were output in the standard VCF format [20]. Because the animals sequenced were categorized as JH1 heterozygotes by genotyping, the candidate variants were filtered to require that each locus was heterozygous for all individuals. Functional annotation of the reduced variant set was performed using SnpEff [21]. Loci were categorized based on *Bos taurus* annotations from Ensembl release 65 for the UMD 3.1 assembly.

### SNP Validation

All SNP and indels within the JH1 candidate interval 15,162,470 to 15,949,175 were subject to repeat mask analysis. Sequences (100 bp flanking) containing the SNP and no repeat sequences were sent to NeoGen-GeneSeek (Lincoln, NE) for sequenome assay design. SNP assay designs were attempted for the flank of each SNP, and bi-directional assays were designed for 14 SNP and only a single assay could be designed for SNP 15438546 (Table 1). Animals for validation genotyping were selected from a DNA archive of animals already genotyped on the Illumina SNP50 for genome selection [22] or as a part of the Bovine HapMap [23]. In all, 768 DNA samples were selected for validation genotyping on a 29-SNP multiplex panel. This set of samples included 8 duplicated samples (16 test DNAs). Twelve samples failed to yield genotypes and 756 samples representing 749 animals had 100% call rate across the 29 assays.

**Table 1:** The 15 SNP in the JH1 suspect region and their locations on the UMD 3.1 assembly.

SNP name	SNP ID	Location (bp)
Hapmap59332-rs29016542	rs29016542	15,162,470
UA-IFASA-2285	rs29012762	15,190,242
Hapmap58058-rs29012133	rs29012133	15,213,319
ARS-BFGL-NGS-10627	rs109677489	15,245,842
ARS-BFGL-BAC-31586	rs108999006	15,305,071
ARS-BFGL-NGS-110213	rs109147458	15,355,777
ARS-BFGL-NGS-10723	rs109495422	15,367,990
Hapmap35322-BES8_Contig457_1759	rs43706895	15,520,367
ARS-BFGL-NGS-17747	rs110050515	15,544,958
Hapmap41817-BTA-29310	rs41565293	15,595,454
ARS-BFGL-NGS-17568	rs109772143	15,656,347
Hapmap35448-SCAFFOLD52197_3147	rs29024927	15,697,628
ARS-BFGL-NGS-119701	rs109320112	15,738,972
ARS-BFGL-NGS-12483	rs109546387	15,908,105
ARS-BFGL-NGS-69055	rs110107689	15,949,175

## Results and Discussion

The initial JH1 interval spanning about 5 Mb on BTA15 from 11,439,502 to 16,147,383 on the UMD 3.1 map [13] was narrowed to a 15-marker window (15,162,470 to 15,949,175) through analysis of additional SNP50 genotypes submitted to the National Dairy Database [24]. In all, the AIPL database contained 23 animals with both the source haplotype and a crossover haplotype that helped this refinement. Also, 34 haplotypes containing the suspect region from the 75-SNP haplotypes were identified in the fine-mapped region, and animals possessing these haplotypes were labeled as carriers. The frequency of JH1 carriers in the analyzed population increased from 21.7% to 23.3% when carriers diagnosed from crossover haplotypes were used in addition to the source haplotype for diagnosis.

Considering the refined area JH1 interval defined by 15 SNP50 markers, 17 crossover haplotypes were detected. The carrier status of animals with these crossover haplotypes was unknown. Only crossover haplotypes that included all of the 15 markers were labeled as carriers, and the remaining haplotypes labeled as non-carriers. Thus, reported JH1 status is conservative, and some heterozygous JH1 animals may have been reported as non-carriers. The true status of these animals could be discovered by breeding trials or by identification of the causative variation through re-sequencing.

Based on this mapping analysis, all heterozygous JH1 animals contained with the CDDR repository were considered as candidates for whole genome re-sequencing. Criteria for selection considered both pedigree relationships between JH1 carriers and non-carrier allele haplotype. The animals chosen for whole genome sequencing included an Observer Chocolate Soldier son (most patriarchal JH1 carrier) and 10 more recent carriers with differing non-carrier haplotype (Table 2). Sequence coverage yields between the 11 animals varied, but were sufficient to identify heterozygous SNP in the JH1 refined interval. Using all data, sequence coverage in this region was quite extensive with 99.93% of all genomic locations covered by greater than three reads (combined samples) and all of the region was covered by at least one read. Additionally, the top 8 samples all covered at least 91% of the region at a read depth of 3 or more (99.2% position with >1 read depth).

**Table 2:** The ten JH1 carrier bulls used for next generation sequencing.

Bull ID^1^	Haplotype^2^	Frequency^3^	Bull Name	Birth Year	Read Depth
8JE0361	45	4.51967	GATES SKY LINE CONGA-ET	1993	9.9
8JE0230	13	2.10855	AMES CHOCOLATE SURVILLE	1978	9.4
14JE0244	8	2.07073	MEADOWAY CHERRY GARCIA	1990	0.6
1JE0655	91	0.69024	WILSONVIEW ARTISTIC ROMEO	2005	5.9
14JE0411	49	0.53896	PEARLMONT HALLMARK CATAMOUNT-E	2002	8.3
8JE0266	14	0.48222	GCG BRASS PRINCE	1985	9.8
200JE0951	38	0.27421	FAIRWAY MORGAN KREMLIN-ET	2004	6.1
11JE0762	80	0.12292	CLOVER FARMS TACO SUPREME	2001	2.7
200JE7039	117	0.06619	ISAU BUSHLEA BROOK BIESTAR	1999	9.1
7JE0657	75	0.01891	GABYS FAIR ROCK-ET	2000	6.9

1 National Association of Animal Breeders (Columbia, MO) identification code.

2 Internal code for unique identification of haplotypes.

3 Haplotype frequency in the population.

Within the original JH1 interval from 11,439,502 to 16,147,383, 20,805 variants were identified that included 17,585 SNPs and 3,220 INDELs. After filtering to retain only heterozygous loci a total of 262 variants remained: 244 SNPs and 18 INDELs. Within the refined JH1 interval, there were only 36 SNP and 2 INDELs. Repeat masking removed 22 SNP and 1 INDEL from the candidate list leaving 15 SNP and one INDEL as potential candidates for the JH1 mutation. Functional annotation identified a single high-impact stop-gain SNP located at position 15,707,169 on BTA15. This C-to-T transition SNP results in an Arginine to a stop codon in exon 3 of *CWC15*, the bovine protein CWC15 homolog of a spliceosome-associated protein [25]. This nonsense mutation would reduce the size of the *CWC15* protein product from 231 amino acids in length to only 54 amino acids. A NCBI conserved domains search on the bovine CWC15 protein product reveals that this truncated protein would not have the conserved Cwf_Cwc_15 (pfam04889) domain present in the wildtype. None of the other 14 SNP or the INDEL fell within the coding regions of the three genes in the refined JH1 interval, but there was one SNP within the 3′ UTR of A7YY77. A Sequenom panel of 29 SNP assays was designed for the validation test [26] based on the 15 SNPs identified by sequence analysis of the refined JH1 interval region (Table S1).

The diagnostic validation of SNP15707169 as the causative mutation was tested against 749 samples. After correcting for one incorrect JH1 assignment due to a crossover in the region, SNP15707169 was 100% concordant with JH1 status based on haplotype (Table S1). Comparing JH1 status to the other 14 SNP loci revealed two other SNP with 100% concordance; however, neither SNP was within the expressed portion of a gene. The SNP calls from all duplicate assays (bi-directional) were in agreement.

Supporting our case for the *CWC15* stop gain SNP as the causative SNP was bovine expression data. Although quantitative expression data for this gene is limited, Harhay and colleagues [27] found *CWC15* was expressed in all 87 bovine tissues supporting its role as an essential gene for cell function. Interestingly, comparison of the expression levels between tissues revealed there was a 7-fold difference normalized tag abundance. Some of the tissues with the highest relative expression of this gene included portions of the placenta and the uterine attachment to the placenta (InnateDB; [28]). *CWC15* is associated in humans with the *PRP19*/*CDC5* complex, which is thought to play an important role in mediating spliceosome activation [29]. Duan et al. [30] have shown that the mouse *CWC15* gene is expressed during early embryonic development. Perhaps a bovine conceptus survives in the absence of functional *CWC15* protein for a short duration until the presence of this gene becomes completely essential for efficient alternative splicing needed for proper development.

For JH1, the causative mutation in *CWC15* is a SNP for which a diagnostic test can easily be developed to identify new carriers. In this study, 730 test results from *CWC15* carriers were used to impute test results for 6,784 animals with 50K genotypes, and concordance was 99.3% between JH1 haplotype status and *CWC15* gene test status. About 1% of the animals reported as free of JH1 were identified as carriers using the imputed SNP genotypes. Other single mutations with additive or recessive inheritance could be imputed using similar methods. The location of the causative mutation –15,707,169 bp – can be used instead of the haplotype to determine carrier status, although detection from haplotypes will continue to be useful until the causative allele is added to SNP chips. Experience with the BovineHD Genotyping BeadChip (Illumina, Inc., San Diego, CA) has shown that having >1,000 animals genotyped for a polymorphism are sufficient to impute genotypes with close to 99% accuracy for >100,000 other animals [16]. Known genotypes for casein variants [31] or diacylglycerol O-acyltransferase 1 [32] for a modest number of individuals could be used to impute missing phenotypes for all other genotyped animals.

Three previously identified genetic defects in Holstein cattle (bovine leukocyte adhesion deficiency (BLAD; [33]), deficiency of uridine monophosphate synthase (DUMPS; [34]), and mulefoot [35]), as well as three deleterious recessives in Brown Swiss cattle (weaver syndrome [36], spinal dysmyelination [37], and spinal muscular atrophy [38]) were each matched to haplotypes using the same automated procedures used to predict JH1. Table 3 lists the conditions, chromosomal positions, number of tested animals, and number of newly identified carriers detected for each of these single-gene traits using March 2012 genotype data. The newly identified carriers have been genotyped but not yet tested for the defect. Results below indicate that official causative mutation or progeny tests would confirm >95% of these haplotype carrier identifications.

**Table 3:** Haplotype vs. official test results for 6 known recessive conditions in Holstein (HO) and Brown Swiss (BS) cattle.

Recessive^1^	Breed	BTA^2^	Location (bp)	Haplotype^3^	Tested animals	Concordance (%)	New carriers
BLAD	HO	1	145,119,004	HHB	11,541	99.9	295
DUMPS	HO	1	69,757,801	HHD	3,060	100.0	3
Mulefoot	HO	15	77,882,504	HHM	87	97.7	111
Weaver	BS	4	∼49,651,768	BHW	7,458	96.3	257
SMA	BS	24	60,148,925	BHM	559	98.0	113
SDM	BS	11	14,742,058	BHD	108	94.4	107

1 BLAD = bovine leukocyte adhesion deficiency; DUMPS = deficiency of uridine monophosphate synthase; Mulefoot = syndactyly; Weaver = bovine progressive degenerative myeloencephalopathy; SMA = spinal muscular atrophy; SDM = spinal dysmyelination.

2 *Bos taurus* (BTA) autosome on which the haplotype is located.

3 HHB = Holstein haplotype associated with BLADS; HHD = Holstein haplotype associated with DUMPS; HHM = Holstein haplotype associated with mulefoot; BHW = Brown Swiss haplotype associated with weaver syndrome; BHM = Brown Swiss haplotype associated with SMA; and BHD = Brown Swiss haplotype associated with SDM.

Haplotype detection is even more accurate if official test results for known mutations are incorporated as an additional SNP within each haplotype. For example, complex vertebral malformation (CVM) [2] could not previously be tracked accurately because two versions of the haplotype exist, one containing and one lacking the causative mutation. Inclusion of the official CVM tests from ancestor bulls allows accurate tracking within the pedigrees of descendants even if they are not tested directly for CVM. However, animals that inherit the equivalent, non-defective haplotype could still be falsely labeled as CVM carriers if pedigree information is missing. Accuracy of carrier status already genotyped animals should also improve if genotypes for the newly discovered mutations are used in addition to the nearby SNP markers.

## Supporting Information

Figure S1 The expected decrease in conception rate for carrier × carrier matings as a function of misidentification rate and carrler frequency
(EPS)

Figure S2 The expected decrease in conception rate for noncarrier × noncarrier matings as a function of misidentification rate and carrler frequency
(EPS)

Table S1 The 15 SNP in the refined JH1 interval region used to design a Sequenom validation test.
(PDF)

Text S1 The effect of pedigree error rates and carrier allele frequencies on the observed reduction in conception rate.
(DOCX)
