# Supplementary material for: Identification of a Nonsense Mutation in CWC15 Associated with Decreased Reproductive Efficiency in Jersey Cattle
Source: PLoS One. 2013 Jan 22;8(1):e54872. doi: 10.1371/journal.pone.0054872 (PMC3551820; doi:10.1371/journal.pone.0054872)
Supplement: Table S1 — The 15 SNP in the refined JH1 interval region used to design a Sequenom validation test. (PDF) [file pone.0054872.s003.pdf]

SnpEff version 2.0.5d (build 2012-01-19), by Pablo Cingolani
Command line: SnpEff eff UMD3.1.65 /media/WorkSpace/JH1/JH1.var.flt.hets.2012-02-06.vcf

# Chromo	Position	Reference	Change	Change_type	Quality	Coverage	Warnings
15	15223488	G	T	SNP	999	70	Repeat
15	15241858	G	C	SNP	999	72	Repeat
15	15242003	A	G	SNP	999	71	Repeat
15	15243503	T	C	SNP	999	75	Repeat
15	15243629	A	G	SNP	999	72	Repeat
15	15243702	T	G	SNP	999	64	Repeat
15	15245529	A	G	SNP	999	92	
15	15246091	C	T	SNP	999	62	Repeat
15	15246682	G	A	SNP	999	83	Repeat
15	15247350	C	G	SNP	999	55	
15	15248938	*	+A	INS	999	80	Repeat
15	15249817	A	G	SNP	999	65	
15	15250807	C	T	SNP	999	77	
15	15261234	G	A	SNP	999	63	Repeat
15	15291915	T	C	SNP	999	80	Repeat
15	15438546	T	A	SNP	999	46	
15	15438555	G	A	SNP	999	50	
15	15441678	C	T	SNP	999	66	
15	15461356	C	T	SNP	999	72	
15	15468272	C	T	SNP	999	74	Repeat
15	15472489	A	G	SNP	999	97	Repeat
15	15473402	G	T	SNP	999	87	Repeat
15	15473477	T	G	SNP	999	67	Repeat
15	15487808	T	C	SNP	999	51	Repeat
15	15572461	C	T	SNP	999	68	
15	15575470	A	G	SNP	999	75	Repeat
15	15581840	G	C	SNP	999	47	
15	15583376	A	G	SNP	999	76	Repeat
15	15585696	A	C	SNP	999	48	
15	15608596	*	+T	INS	90.3	98	Repeat
15	15668397	T	C	SNP	999	70	
15	15668855	T	C	SNP	999	84	Repeat
15	15668875	A	G	SNP	999	86	Repeat
15	15687701	A	G	SNP	999	76	Repeat
15	15694793	C	T	SNP	999	84	Repeat
15	15703397	C	A	SNP	999	55	
15	15707169	C	T	SNP	999	71	
15	15715994	A	C	SNP	999	80	

Assays Designed	JH1 Concordance	Gene_ID	Gene_name
0			
0			
0			
0			
0			
0			
2	99.7%		
0			
0			
2	99.7%		
0			
2	99.1%		
2	99.7%		
0			
0			
1	29.3%		
2	95.1%		
2	95.4%		
2	99.5%		
0			
0			
0			
0			
0			
2	100.0%		
0			
2	100.0%	ENSBTAG00000024803	A7YY77_BOVIN
0		ENSBTAG00000024803	A7YY77_BOVIN
2	95.2%	ENSBTAG00000024803	A7YY77_BOVIN
0		ENSBTAG00000024803	A7YY77_BOVIN
2	45.2%	ENSBTAG00000001695	ENSBTAG00000001695
0		ENSBTAG00000001695	ENSBTAG00000001695
0		ENSBTAG00000001695	ENSBTAG00000001695
0			
0			
2	93.9%	ENSBTAG00000007913	CWC15_BOVIN
2	100.0%	ENSBTAG00000007913	CWC15_BOVIN
2	96.1%	ENSBTAG00000007913	CWC15_BOVIN

Bio_type	Trancript_ID	Exon_ID	Exon_Rank
protein_coding	ENSBTAT00000034535		
protein_coding	ENSBTAT00000034535		
protein_coding	ENSBTAT00000034535		
protein_coding	ENSBTAT00000034535		
pseudogene	ENSBTAT00000002224		
pseudogene	ENSBTAT00000002224		
pseudogene	ENSBTAT00000002224		
protein_coding	ENSBTAT00000010403		
protein_coding	ENSBTAT00000010403	exon_15_15707138_15707250	3
protein_coding	ENSBTAT00000010403		

Effect	old_AA/new_AA	Old_codon/New_codon
INTERGENIC		
INTERGENIC		
INTERGENIC		
INTERGENIC		
INTERGENIC		
INTERGENIC		
INTERGENIC		
INTERGENIC		
INTERGENIC		
INTERGENIC		
INTERGENIC		
INTERGENIC		
INTERGENIC		
INTERGENIC		
INTERGENIC		
INTERGENIC		
INTERGENIC		
INTERGENIC		
INTERGENIC		
INTERGENIC		
INTERGENIC		
INTERGENIC		
INTERGENIC		
INTERGENIC		
INTERGENIC		
INTERGENIC		
DOWNSTREAM: 3183 bases		
DOWNSTREAM: 1647 bases		
UTR_3_PRIME: 2300 bases from CDS		
INTRON		
DOWNSTREAM: 4006 bases		
DOWNSTREAM: 3548 bases		
DOWNSTREAM: 3528 bases		
INTERGENIC		
INTERGENIC		
UPSTREAM: 2063 bases		
STOP_GAINED	R/*	Cga/Tga
DOWNSTREAM: 2042 bases		

Codon_Num(CDS)	Codon_Degeneracy	CDS_size
		1503
55	1	696
