# Supplementary material for: Identification of a Nonsense Mutation in CWC15 Associated with Decreased Reproductive Efficiency in Jersey Cattle
Source: PLoS One. 2013 Jan 22;8(1):e54872. doi: 10.1371/journal.pone.0054872 (PMC3551820; doi:10.1371/journal.pone.0054872)
Supplement: Text S1 — The effect of pedigree error rates and carrier allele frequencies on the observed reduction in conception rate. (DOCX) [file pone.0054872.s004.docx]

Let m be the misidentification rate for both sires and MGS and let p be the carrier frequency. If the pedigree sire is a carrier, the true sire is a carrier with Prob = (1 - m) + mp, and if the pedigree sire is a noncarrier, the true sire is a noncarrier with Prob = (1 - m) + m(1 - p) = 1 - mp. The same is true for MGS. For carrier × carrier matings the observed loss will be:

L_O_ = (1 - m + mp)^2^ × L_E_

where L_O_ and L_E_ are the observed and expected losses, respectively. The decrease in L_O_ is more sensitive to changes in misidentification rate than changes in carrler frequency (Figure S1).

For noncarrier × noncarrier matings, some will actually be carriers, causing the observed 'normal' mean to increase by:

L_O_ = (mp)^2^ × L_E_

These matings will result in an increase in L_O_ only when misidentification rates and carrier frequencies are high (Figure S2). The observed loss can be approximated as L_O_ = (1 - m)^2^ × L_E_ if p is fairly small.

If the JH1 expected loss is 4.6% and the misidentification rate is 10%, which is consistent with literature estimates, the observed loss would decrease to (1 - 0.1)^2^ × 4.6% = 3.7%, which is identical the results presented in the paper.
